# Treatment of Hysterical Attacks

**Published:** 1875-12

**Authors:** 


Treatment of Hysterical Attacks.—According to M. Charcot, who has lauded this very highly, compression made with the hands over the abdominal region corresponding to the ovaries, will almost instantly arrest the most violent attack of hysteria. In this regard Dr. Caffe reports, in his journal, the following interesting facts: More than thirty-five years have passed since I witnessed, with Professor Chomel, a most violent attack of hysteria in a young woman of high social position, thwarted in her marriage projects. The very learned clinician (who was at the same time family physician) immediately advised me to make compression with both hands in the iliac fossæ of the patient. The attack promptly subsided through the compression of the aura hysterica.—*La Tribune Med.*

